# Lead Palsy Treated with Iodide of Potassium

**Published:** 1853-12

**Authors:** James M. Newman

**Affiliations:** Buffalo, N. Y.


ART. II.—*Lead Palsy treated with Iodide of Potassium.* Reported to the Buffalo Medical Association, November 1, 1853. By JAMES M. NEWMAN, M. D., Buffalo, N. Y.

*June 6th*, 1853. Mr. O'C., aged 30 years, a painter by trade, applied to me for treatment. Has worked at his business between seven and eight years. His health has been poor for a year past, so as to compel him to cease from work at times, and he lost, in consequence, the greater part of the winter. For the last three weeks has been obliged to lie idle, from severe pain in both shoulders and arms, with loss of power in them. He can with difficulty raise them above his head. A dirty, dark blue line, encircles the gums and teeth in both the upper and lower jaws. His bowels are costive, appetite poor, complexion sallow, and general appearance indicative of depraved health. In person and dress he is extremely uncleanly.

I directed

℞ Iodid. Potass. ℥ss.
Decoct. Sarsap. ℥xvi.

Dose, *two teaspoonfuls four* times a day. M.

℞ Pill, cathar. comp. No., 1 every other night.

11*th*. No apparent improvement. Pain in arms last night "more severe than ever."

℞ Tinct. aconit.
Tinct. arnica, $\overline{aa}$ ℥iv.

Bathe arms two or three times a day. M. ft. liniment.

Continue iodid. potass.

14*th*. Not much perceptible improvement. Directed him to increase the dose of iodide of potassium to *three tablespoonfuls three* times a day.

Continue the use of the liniment.

15*th*. Feels better; pain not so severe. Appetite good.

24*th*. Improving. Pain much reduced, and what little remains has transferred itself to just above the wrists. Appetite good, bowels regular and the deep blue color of the gums is lightening up, though still too dark by far to be healthy.

He has taken since commencement of treatment on the 6th instant, iodid potass., ℥ss.

To day directed ℞ Iod. potass., ℥ss.

Tinct. iodine, ʒij.

Aqua, Oj. M.

Dose, three tablespoonfuls *three* times a day.

*July 6th*. Improvement slow but apparent. Gave to day

℞ Iod. Potass., ℥j.
Aqua, Oj. M.

Take ʒvi. (by actual measurement,) three times a day in water.

*Notes.*—The above will give 67½ grains hydr. potass. per day.

19*th*. Finished the above prescription on the 17th inst.

Pain has nearly all left his arms, but they are yet powerless, or "quite dead," as he terms it. General health and appearance improved.

℞ Iodid. potass., ℥j.
Aqua, ℥xvi.

Dose, ʒvi. three times a day.

Also, ℞ Acetic solution strychnine, ℥ss., (containing one grain strych. to the ounce.)

Dose, 15 drops three times a day.

*Aug.* 10*th*. Finished his last prescription yesterday. Has not taken his medicines with as much regularity as previously, but reports himself much better. The pain has entirely left his arms, and their power is returning.

℞ Sol. strych., ℥ss.

Dose and frequency same as before.

℞ Iod. potass., ℥j.
Aqua, Oj.

Dose, ʒvi. three times a day.

*Sept.* 13*th*. Met him in the street. Considers himself well. Has taken no medicine for a week past.

O'C. has taken during the above period of treatment, iodid. potass. ℥iv., or 1920 grains.

